# Antimicrobial activity of the *trans-cinnamaldehyde *on nosocomial enteric bacilli producers of extended spectrum β-lactamase (ESBL)

**DOI:** 10.1186/1753-6561-8-S4-P89

**Published:** 2014-10-01

**Authors:** Vicente Pinto, César Barbosa, Pedro Magalhães, Camila Coelho, Joseires Fontenelle, Gerardo Cristino-Filho, Helliada Chaves, Antonio Silva, Alrieta Teixeira, Mirna Bezerra

**Affiliations:** 1 Faculty of Medicine of Sobral, Master's Program in Biotechnology, Federal University of Ceará (UFC), Campus-Sobral, Ceará, Brazil; 2 Faculty of Dentistry of Sobral, Federal University of Ceará (UFC), Campus-Sobral, Ceará, Brazil; 3 Department of Physiology and Pharmacology, Faculty of Medicine, Federal University of Ceará (UFC), Fortaleza, Ceará, Brazil; 4 Faculty of Medicine of Sobral,Federal University of Ceará (UFC), Campus-Sobral, Ceará, Brazil; 5 Masters in Biotechnology, Federal University of Ceará (UFC), Campus-Sobral, Ceará, Brazil

## Background

The extended spectrum ß-lactamases (ESBL) are enzymes that produce resistance to ß-lactam antibiotics, including penicillins, wide spectrum cephalosporins and aztreonam, by cleavage of ß-lactam ring (BUSH & JACOBY, 2010). Since ESBL-producing bacteria are frequently associated with nosocomial infections, treatment options are becoming increasingly limited (RAWAT & NAIR, 2010). In this context, the discovery of compounds which can inhibit the growth of micro-organisms which produce these enzymes becomes increasingly important.

## Methods

In this study were evaluated the antimicrobial activity of *trans-cinnamaldehyde *by microdilution technique and also determined its minimum bactericidal concentration (MBC) on nosocomial enteric bacilli ß-lactamases producers. We analyzed 45 bacterial species, 36 to the *Enterobacteriaceae *family and nine of the other species of Gram-negative bacteria. The most prevalent species ESBL-producing was *Klebsiella pneumoniaessp. pneumoniae *(70% of isolates of this specie). The detection of ESBL was performed by phenotypic testing (approximation discs, combination discs and minimum inhibitory concentration - MIC - using E-test).

## Results and conclusions

The *trans-cinnamaldehyde *showed antibacterial activity and promote inhibition of growth for all planktonic microorganisms ESBL positive tested, with MIC ranging between 0.95 mM and 1.90 mM. Bactericidal activity was detected at a concentration of 1.90 mM, regardless of the species analyzed in this study. Our results suggest that *trans-cinnamaldehyde *is a compound with potential antimicrobial against ESBL-producing bacteria and can be employed both in preventing infection through their application in solutions used in the processes of disinfection of hospital instruments and equipment but also in drug development for topical action.
